# Editorial: Chronic stress, telomeres and aging

**DOI:** 10.3389/fendo.2024.1504405

**Published:** 2024-11-18

**Authors:** Gabriele Saretzki

**Affiliations:** Biosciences Institute, Newcastle University, Newcastle upon Tyne, United Kingdom

**Keywords:** chronic stress, telomeres, aging, inflammation, endocrinology

In the journal “Frontiers in Endocrinology”, subheading “Endocrinology of Aging” a research focus was initiated on the Research Topic “*Chronic stress, telomeres and Aging*” with 3 associated guest editors: Theologos M Michaelidis (University of Ioannina, Greece), Gabriele Saretzki (Newcastle University, UK) and Jue Lin (University of California, San Francisco, USA).

During 2023 and 2024 5 papers were published on this Research Topic on a diverse content: 3 of them included research on telomeres and 2 dealt with aspects of inflammation during aging and in specific age-related diseases.

The first paper was “*Association between telomere length and erectile dysfunction: A cross-sectional study*” by Chen et al. from Fujian Medical University Union Hospital in China. The aim of the study was to explore whether there is an association between age-related erectile dysfunction (ED) and leukocyte telomere length (LTL). To achieve this, the authors used data from the National Health and Nutrition Examination Survey (NHANES, USA) with a sample size for this study of 1,694 individuals from 20 years of age onwards. Weighted multivariate regression analyses revealed shorter LTL in people with ED compared to subjects without the condition. The authors explained this result with a number of different co-morbidities as well as chronic stress conditions, the topic of our Research Topic, in patients with ED. In addition, other statistical methods found a non-linear relationship between LTL and ED and defined further subgroups. In summary, the study found that individuals with longer LTL have a lower risk of developing ED. Consequently, the authors suggest that preventing telomere shortening by medical interventions could delay the onset as well as the risk of ED in middle-aged men. One could add here that also changes in lifestyle such as not smoking, healthy nutrition and regular exercise might be able to help to prevent conditions of chronic and oxidative stress which are known to be inversely correlated to telomere length in lymphocytes and thus result in diseases such as cardiovascular disease, hypertension, and obesity (1–3).

Another clinical topic regarding telomere shortening was raised by a Japanese research team. Their study is titled “*Accelerated telomere shortening in adrenal zona reticularis in patients with prolonged critical illness*“ by Nonaka et al., from the Tokyo Metropolitan Institute for Geriatrics and Gerontology. The authors analysed the association between prolonged critical illness (PCI), which certainly can be counted as a serious chronic stress, and telomere lengths in human adrenal cells. Telomere shortening has been shown to accelerate under physiological as well as psychological stresses *in vitro* as well as *in vivo* (4, 5). For this, adrenal glands from 18 patients who died after PCI were compared to those from 14 normal subjects who died of unrelated causes. The human adrenal gland plays an important role as the final organ of the hypothalamic–pituitary–adrenal axis since it maintains homeostasis under various conditions, including chronic diseases such as PCI. The adrenal gland consists of two separate regions: the cortex and the medulla. The cortex produces steroid hormones, while the medulla consisting predominantly of chromaffin cells, produces catecholamines. The adrenal cortex has three morphologically and functionally defined zones: the outer zona glomerulosa (ZG) which produces mineralocorticoids, the middle zona fasciculata (ZF) producing glucocorticoids (cortisol) and the inner zona reticularis (ZR) that synthesises adrenal androgens (DHEA). Using quantitative fluorescence *in situ* hybridization (qFISH) the authors determined relative telomere lengths (RTLs) in the parenchymal cells of the three adrenocortical zones and in the chromaffin cells of the medulla. Intriguingly, telomere length in cells from the zona reticularis was highly significantly shorter in the PCI group than in the control group for both men and women. In contrast, RTL in the other 3 adrenal cell types was not different between controls and PCI cases. In addition, it was found that numbers and proliferation of cells in the ZR were higher in the PCI group. The authors interpret this observation as a reactive, disease-induced proliferation which could underlie the accelerated telomere shortening and aging process in this adrenal area. This means, by analysing telomere length, the authors were able to pinpoint subtle changes in the morphology and functionality of specific areas of the adrenal gland which correlated to an increased chronic stress due to various clinical illnesses.

The third paper involving telomeres relates to an animal population: “*Birds of a feather age together: Telomere dynamics and social behaviour predict life span in female Japanese Quail (Coturnix japonica)*“ from McCollum et al. from the Bucknell University in Lewisburg, United States. Their study analyses chronic social stress as a potential link between social behaviour and a healthy lifespan in their animal model. Social stressors can trigger the vertebrate endocrine stress response by releasing catecholamines and glucocorticoids in order for individuals to respond to different challenges. While these hormones enable individuals to survive short-term stressors, an extended exposure to glucocorticoids is associated with increased levels of oxidative stress for cells, cardiovascular disease and even increased mortality risk. Telomeres are particularly sensitive to oxidative stress and thereby function as sentinels for increased stress. It is well known that chronic oxidative as well as social stress can lead to accelerated telomere shortening and increased aging rates in humans as well as model organisms (6, 7). Social life can both induce stress but also help to combat it. Japanese quail live in social group with a dynamic hierarchy which is dependent on the environment (food supply etc.) as well as inter-individual interactions to maintain or change the social hierarchy. The study analysed social support as well as anti-social behaviour in these social birds which were kept and observed in permanent groups. The different behaviour types were correlated to physiological measures such as changes in telomere length, corticosterone levels as well as lifespan. The study found that birds with a high degree of social support (affiliate behaviour) in their groups had no changes in basic glucocorticoid hormone levels which correlated to longer telomeres and a lower telomere shortening rate over time and they also lived longer. In contrast, birds which were under a high amount of agonistic behaviour had shorter telomeres, higher stress levels and lived shorter. Interestingly, the authors also suggested that affiliative behaviour may have a genetic component (McCollum et al.). In addition, social support is also able to decrease inflammation as well as oxidative stress (9), both resulting in shorter telomeres (10). Like in most higher vertebrate species, telomere length decreased over the lifetime of the quails. Intriguingly, birds with a lower rate of telomere loss lived substantially longer (up to 3.5 years) while those with high telomere shortening rates died as early as one year. Similar correlations between telomere shortening and longevity were published previously for humans (11). Interestingly, telomere shortening rates at one year of age in the quails predicted longevity of the birds. This means that early life events and experiences can have long-term effects on levels of cellular damage and organismal lifespan (McCollum et al.). However, affiliate behaviour and beneficial social interactions can have also additional benefits on other physiological parameters than telomere length. Here, glucocorticoid levels, including that of the mothers, might play a role for the type of social behaviour individuals prefer and determine resilience against aggressiveness (McCollum et al.). Moreover, others had shown previously that high corticoid levels can also translate into more oxidative stress and damage (12). However, due to varying glucocorticoid levels during lifetime, more detailed studies are required for a better understanding of the relationship between stress hormones, cellular damage, oxidative stress and longevity. This underlines the importance of our selected Research Topic for endocrinology on chronic stress, telomeres and aging. The authors emphasise the beneficial effect of positive social interactions which can also be translated into human conditions since it is already known that in humans, high levels of social support associate with better age-related outcomes, including reduced rates of cancer, increased immunity, and longer lifespan (8).

In addition to telomere shortening, aging is also characterised by chronic inflammation. In fact, a previous study identified inflammation as the most important predictor of longevity in human centenarians (13). This Research Topic, specifically related to a condition of hypogonadism was at the centre of the review “*A Narrative Review on Inflammaging and Late-onset Hypogonadism*“ from Xing et al. from the Andrology Department of Integrative Medicine, at the Zhongda Hospital of the Southeast University in Nanjing, China. The authors argue that increasing human life expectancy achieved during the last decades also correlates to more diseases such as late-onset hypogonadism (LOH) in older men. This condition is linked to a decline in testosterone levels and impacts both mental and physical well-being of these men. Recently, some interest was raised on the influence of inflammation and, in the context of ageing, inflammaging in the development of this condition. The concept of inflammation was developed by Claudio Franceschi from the University of Bologna in Italy (14). Aging is characterised by a chronic, low-grade systemic inflammation that can influence longevity in addition to telomere length while both factors also interact (10). Among the underlying mechanisms for the aging process various contributors have been considered, including senescence of cells and the immune system, telomere shortening, mitochondrial dysfunction as well as defects in autophagy and proteostasis (15, 16). The aim of the above review was to discuss the potential influence of these processes onto the development of late-onset hypogonadism and to suggest possible interventions to counteract inflammaging in order to develop new treatment strategies for the condition (Xing et al.). As Leydig cells in male gonads produce less testosterone with age, they experience higher levels of oxidative stress that disrupts signalling pathways for testosterone production and Leydig cell function. In addition, the cells also experience an increased low-grade inflammation with an increased production of various pro-inflammatory cytokines such as C-reactive protein (CRP), IL-6, TNFα and IL-1β (17). These factors are predominantly activated by inflammatory signalling pathways involving NF-KB and P38MAPK that play an important role in aging. Strikingly, due to the lack of a protective blood-testis barrier, these inflammatory cytokines are able to enter the testicular interstitium from the circulation and adversely affect Leydig cell function (18). The authors present various possible therapeutic strategies for the treatment of late-onset hypogonadism, mainly focusing on combating inflammatory processes in the body. These include physical exercise, cell transplantation, anti-inflammatory and antioxidant drugs as well as traditional medicine approaches (Xing et al.).

The second paper on the role of inflammation in an age-related disease was an original study: “*Causal roles of circulating cytokines in sarcopenia-related traits: a Mendelian randomization study*“ by Chen et al., from Fujian Medical University Union Hospital in China. Like for hypogonadism, inflammation is also tightly connected to sarcopenia-the loss of muscle mass and strength in older people resulting in an increased risk of falls and hospitalisation. Chronic inflammation, be it local or systemic, can change the function of immune cells and with it also the balance between pro-and anti-inflammatory cytokines and molecules. However, various studies reached partly contradictory data, for example, describe either an increase or a decrease in the pro-inflammatory cytokine IL-6. In addition, known major cytokines such as TNFα and CRP seem to be involved in age-related processes such as decreased grip-strength (19), but again, contradictory data exist here. These apparently inconsistent data on various inflammatory cytokines involved in the condition might be due to small numbers of subjects in various studies, a rather limited set of cytokines being included or the involvement of several confounding factors. The current study performed a two-sample Mendelian randomization (MR) analysis from almost 9000 subjects in order to establish a causality between a substantial number of circulating cytokines and features of sarcopenia (Chen et al.). The analysis used genetic data on 41 different cytokines from genomic association studies (GWAS) as well as physiological data such as grip strength and walking pace, often also analysed for frailty traits. The study found 3 specific cytokines such as hepatocyte growth factor (HGF), interferon gamma (IFNγ)-induced protein 10 (IP-10) (also known as CXCL-10) and macrophage colony-stimulating factor (M-CSF) that correlated well with appendicular lean muscle mass and thus might play a protective role against sarcopenia. Importantly, HGF is known to be involved in the proliferation and differentiation of muscle satellite cells and thus regulates skeletal muscle tissue regeneration via the mobilization and modification of bone marrow stem cells. CXCL-10 is involved in T-cell activation and migration but was recently also found in the regulation of myogenic differentiation of myoblasts *in vitro* (20), but again, contradictory results have been found in other studies. M-CSF is a monocyte mobilizer involved in survival, proliferation and maturation of macrophages and was used successfully in mouse models of muscle regeneration after injury. On the other hand, 3 other factors such as interleukin-7 (IL-7), monocyte chemotactic protein 3 (MCP-3), and regulated on activation, normal T cell expressed and secreted (RANTES) were found to have increased levels and correlated with a decreased grip strength. IL-7 is a muscle-specific cytokine implicated in the myogenesis and migration of skeletal muscle cells. MCPs are chemotactic cytokines with MCP-1, a related family member, having an apparent age-related increase in rodents and humans accelerating muscle catabolism and consequently impairing muscle strength. RANTES is involved in the aggregation of inflammatory cells and persistent inflammatory response as well as implicated in muscle-wasting diseases, such as Duchenne muscular dystrophy and accumulated in skeletal muscles of sarcopenic rats. Thus, the authors defined and confirmed 6 different cytokines that can be changed during sarcopenia. A better knowledge of important inflammatory cytokines that are involved in the development of age-related sarcopenia might result in novel therapeutic options for this age-related disease.

In conclusion, all 5 published papers shed new light on the interplay of chronic stress, endocrinological factors as well as genetic elements such as telomeres that are known as being sensitive sensors of oxidative stress and DNA damage and the involvement of these players in the aging process.
